# Stone in the Bladder

**Published:** 1876-08

**Authors:** J. W. S. Gouley

**Affiliations:** Professor of Diseases of the Genito-Urinary System, in the Medical Department of the University of the City of New York, Surgeon to Bellevue Hospital, etc.


ATLANTA
MEDICAL AND SURGICAL JOURNAL

VOL. XIV.] AUGUST—1876. [No. 5

### Original Communications.

### STONE IN THE BLADDER:

### ITS SPONTANEOUS EXPULSION AND ITS REMOVAL BY LITHOTRIPSY, LITHOTOMY AND PERINEAL-LITHOTRITY, WITH AN ANALYSIS OF THIRTY-FIVE CASES; BEING AN ABSTRACT OF A PAPER READ TO THE MEDICAL SOCIETY OF THE STATE OF NEW YORK, JUNE, 1876.

J. W. S. GOULEY, M.D.,
Professor of Diseases of the Genito-Urinary System, in the Medical Department of the University of the City of New York, Surgeon to Bellevue Hospital, etc.

An abstract only of the paper is here given, owing to its length, and because it will appear in full elsewhere. It contains a brief account of the genesis of vesicle stones, of the treatment of nephritic colic, of the use of alkalies and other remedies in expediting the spontaneous expulsion of small calculi from the ureters and from the bladder, of the early detection of stone, and of the instruments best adapted to the purpose.

The writer considers that the time has arrived for lithotripsy to be adopted as the general operation in adult males whose urethræ are normal or can be rendered so practically; for lithotomy to be resorted to only in cases accompanied by narrow and undilatable urethral strictures, or by cystitis with uncontrollable vesical irritability, and in young children; and for perineal-lithotrity and its modifications to be reserved for cases of large stones.

A succinct account of these various modes of treatment of stone in the bladder is followed by a report of thirty-five cases, an analysis of which is here given.

The thirty-five patients suffering from vesical calculi, who had come under treatment, ranged from two to seventy-four years of age.

In seven cases the stones were expelled spontaneously from the bladder.

Thirteen cases were subjected to lithotripsy.

Ten cases underwent lithotomy, and in five cases perineal-lithotrity was done. Thirty-three recovered, and two died.

The seven patients whose calculi were expelled from the bladder were from twenty-five to sixty-two years of age.

They had all had from one to four attacks of nephritic colic, and five had already expelled calculi per urethram.

Two had urethral strictures; in one of these two calculi were impacted behind a stricture which had to be dilated before they could be extracted.

Two had prostatic hypertrophy, but the stones were finally expelled.

In one case the calculi were very small but numerous.

In one case there were ten calculi the size of buckshot.

In one case there were four, one of which was equal in diameter to No. 15 sound, and weighed ten grains.

In one case there were two, both of which became impacted behind a urethral stricture, and were extracted with dressing forceps.

There was one in each of the three remaining cases, and in one of these cases the stone weighed ten grains.

The calculi were all of uric acid, and were expelled from the bladder in from one to thirty days or more after the nephritic colic.

The treatment consisted in the use of diluents and alkalies, cathartics, dilatation of the urethra, etc.

All the patients made good recoveries.

The thirteen patients subjected to lithotripsy were between the ages of 24 and 66; three were under 30; three were from 34 to 37; three were from 48 to 60, and four from 62 to 66.

Eight of the thirteen had troublesome cystitis, with much vesical and urethral sensitiveness, which required prolonged treatment before the operation could be done.

Three had urethral strictures, which, however, yielded to gradual dilatation before the operation.

In five cases it was necessary to enlarge the meatus urinarius by incision.

Four cases were complicated with prostatic enlargement.

Two cases had before undergone lithotomy.

Two cases had already been lithotriptized.

The stone was small in two cases; of medium size in five cases, and exceeds one inch in diameter in four cases.

There were several stones in one case; four in one case; two exceeding one inch in diameter in one case, and one stone in each of the ten remaining cases.

Every case underwent the most careful preparatory treatment.

Four cases were cured in one sitting; one in three sittings; one in four sittings; four cases in five sittings each; one case in seven sittings, and one in twenty-four sittings.

One case was followed by a rigor after the first sitting; one case after each of the four sittings; while the remainder had no rigors at all.

In one single case did any of the sittings exceed five minutes, and that by one minute only. In most of the cases the sittings were of from one and a half to three minutes' duration.

The lithotrite was introduced twice in one sitting in three cases. In all the others the instrument remained in the bladder until from three to fifteen crushings had been made when it was withdrawn not to be again used until the next sitting.

The intervals of time between the sittings varied from two to eight days, except in two cases, where they were extended to several months at the request of patients.

In no case did orchitis supervene.

No sitting has been followed by severe hemorrhage, and only in some of the patients of sixty and upwards has the urine been tinged with blood. Even in these cases the urine became clear within twenty-four hours.

No sitting has been followed by severe cystitis, on the contrary, after the second sitting the vesical irritability has usually so diminished as to render the subsequent sittings easy for the patient and for the operator.

Expulsion of detritus from the beak of the lithotrite was always effected to a sufficient extent to permit closure of the jaws, so that the urethra sustained no injury in any case during the withdrawal of the instrument. The detritus was spontaneously expelled with the urine in ten cases. Aspiration of the fragments was made in one case. In one case aspiration was used only during one sitting, the detritus having been expelled spontaneously afterwards. In one case all the detritus was removed by the lithotrite and by an evacuating catheter, as none could be expelled on account of vesical atony. Impaction of fragments in the urethra occurred in five cases, but caused little inconvenience in any case, as they were either very soon expelled or forthwith extracted. The amount of detritus gathered varied from a few grains to an ounce and a quarter. The most rigid after treatment was enjoined in each case.

There has been no recurrence of the trouble in any case except one, in which calculi were periodically escaping from the ureters. This was the fatal case. One patient died, and the cause of his death was pyelonephritis, both kidneys and ureters being filled with small calculi. The operation had to be discontinued twice on account of ths renal trouble, and death occurred about eight weeks after the suspension of the treatment.

Of the ten patients upon whom lithotomy was performed, one was twenty-four years old, and the others were all under ten years of age. The eldest of these was eight years, the youngest two years of age.

Three patients had troublesome cystitis.

One had urethral stricture.

The stone was large in one case, in the others it was of medium size and small.

There were three stones in one case; two in another, and one in each of the remaining eight cases.

Lateral perineal cystotomy was done in five cases.

Lateral section of the anterior half of the prostate with cystectasy, was done in three cases.

The median operation was performed in two cases.

Fragmentation of the stone was necessary, before extraction, could be accomplished, in one case, and in this lateral perineal cystomy was done. Extraction was not attended with difficulty in any of the other cases.

There was no serious hemorrhage in any case.

The urine was passed at will, from the day of the operation, in four cases.

The urine flowed entirely through the urethra, from the eleventh day after the operation, in one case; from the eighth day in one case; from the fourth day in one case; time not stated in five cases.

In two cases all the urine, from the time of the operation, was passed at will, not a drop escaping by the wound. The wound was healed in twenty-eight days in one case; in twenty days in two cases; in fourteen days in three cases; in ten days in two cases; in seven days in one case; and the date of union of the wound was not stated in one case.

In one case a fistula still exists, through which a few drops of urine escape.

All the patients recovered.

Of the five patients subjected to perineal-lithotrity, one was seventeeen, one thirty-seven, one sixty, one seventy-four, and one fifty-two years of age. One had been lithotomized seven years before. They had all long been distressed with cystitis. In one case there were two stones, the four others had only one calculus each. In one case the nucleus of the stone was a piece of glass.

In all the cases the stones were large, the detritus weighing in Case I., twelve hundred grains; in Case II., four hundred and eighty grains; in Case III., two hundred and seventy grains; in case IV., nine hundred and sixty grains; in Case V., four hundred and nine grains. They consisted all mainly of phosphates.

The perineal incision was made freer than usual, and the bulb of the urethra had to be divided for nearly its whole length in one case. (Case I.)

In two cases the incision was made in accordance with Dolbeau's directions, and the membranous portion of the urethra only was divided.

In two cases the bistoury was plunged, cutting edge upwards, into the median line of the perinæum, close to the anal margin, until its point entered the groove of the staff, and nearly the whole membranous portion of the urethra cut longitudinally from behind forwards, and the cutaneous incision was completed at one sweep in withdrawing the knife.

In one case, dilatation of the prostatic urethra and neck of the bladder was made partly with a two-bladed dilator, partly with the finger. Dolbeau's six-branched dilator was used in three cases, and the hydraulic dilatator in one case.

Fragmentation was made partly with a fenestrated lithotrite, introduced through the normal route, partly with strong forceps passed through the artificial opening, in two cases. Dolbeau's and my own lithoclasts were used in the others.

The stone was hard in one case, and soft in four cases.

Exploration, as recommended by Dolbeau, with small lithotomy forceps, was made in all the cases, and in three instances the stone, being once seized, could not without difficulty, and twice not without considerable delay, be disengaged from the forceps.

Extraction of the detritus was, in all the cases, effected with the lithoclasts, with lithotomy forceps and with the scoop.

In all the cases frequent vesical injections of cold water had the happy effect of bringing within reach large and small fragments, after the bladder had seemed free from calculous matter.

Contrary to Dolbeau's directions, the finger was a number of times introduced into the bladder in every case without giving rise to the least untoward symptom.

The loss of blood did not exceed four ounces in any case.

In one case the urine was passed at will from the day of the operation; in one case within twenty-four hours thereafter; and in one case in forty-eight hours. In one case the urine dribbled from the wound for two weeks. In the fatal case the urine also dribbled from the wound, very little occasionally passing through the normal route.

The wound healed in two weeks in two cases. A perineal fistula was established as a precautionary measure against cystitis in one case.

Four patients recovered and one died in a week, of pyæmia.

